# Immunization with a streptococcal multiple-epitope recombinant protein protects mice against invasive group A streptococcal infection

**DOI:** 10.1371/journal.pone.0174464

**Published:** 2017-03-29

**Authors:** Chih-Feng Kuo, Nina Tsao, I-Chen Hsieh, Yee-Shin Lin, Jiunn-Jong Wu, Yu-Ting Hung

**Affiliations:** 1 Department of Nursing, College of Medicine, I-Shou University, Kaohsiung, Taiwan; 2 Department of Biological Science and Technology, College of Medicine, I-Shou University, Kaohsiung, Taiwan; 3 Department of Microbiology and Immunology, College of Medicine, National Cheng Kung University, Tainan, Taiwan; 4 Department of Medical Laboratory Science and Biotechnology, College of Medicine, National Cheng Kung University, Tainan, Taiwan; 5 Department of Biotechnology and Laboratory Science in Medicine, School of Biomedical Science and Engineering, National Yang-Ming University, Taipei, Taiwan; Instituto Butantan, BRAZIL

## Abstract

*Streptococcus pyogenes* (group A *Streptococcus*; GAS) causes clinical diseases, including pharyngitis, scarlet fever, impetigo, necrotizing fasciitis and streptococcal toxic shock syndrome. A number of group A streptococcus vaccine candidates have been developed, but only one 26-valent recombinant M protein vaccine has entered clinical trials. Differing from the design of a 26-valent recombinant M protein vaccine, we provide here a vaccination using the polyvalence epitope recombinant FSBM protein (rFSBM), which contains four different epitopes, including the fibronectin-binding repeats domain of streptococcal fibronectin binding protein Sfb1, the C-terminal immunogenic segment of streptolysin S, the C3-binding motif of streptococcal pyrogenic exotoxin B, and the C-terminal conserved segment of M protein. Vaccination with the rFSBM protein successfully prevented mortality and skin lesions caused by several *emm* strains of GAS infection. Anti-FSBM antibodies collected from the rFSBM-immunized mice were able to opsonize at least six *emm* strains and can neutralize the hemolytic activity of streptolysin S. Furthermore, the internalization of GAS into nonphagocytic cells is also reduced by anti-FSBM serum. These findings suggest that rFSBM can be applied as a vaccine candidate to prevent different *emm* strains of GAS infection.

## Introduction

Group A streptococcus (GAS) is an important human pathogen, and it causes a variety of diseases ranging from noninvasive pharyngitis to invasive diseases, such as necrotizing fasciitis, sepsis, and streptococcal toxic shock syndrome. GAS also causes nonsuppurative complications, including acute glomerulonephritis and rheumatic fever. The number of clinical invasive GAS cases has increased worldwide over the past twenty years [1–6]. The burden of GAS invasive diseases was estimated in developed countries as 17.8 million new cases per year, and at least 517,000 deaths per year caused by severe GAS infection and its severe complications have been reported [6]. Although several vaccine candidates against GAS infection have been developed, only one 26-valent recombinant M protein vaccine has achieved success in a class II clinical trial [7–9]. M protein is encoded by the *emm* gene and more than 220 *emm* types have been reported [10]. The most prevalent *emm* genotypes associated with invasive GAS infection are *emm1*, *emm3*, *emm4*, *emm6*, *emm12*, *emm28*, and *emm89* in Canada, the United States, and European countries [1–3, 5]. In contrast, a higher diversity of *emm* types has been found in Africa and Pacific regions [4, 6]. In Taiwan, the leading *emm* types of GAS isolated from invasive infection are *emm1*, *emm4*, *emm11*, *emm12*, and *emm102* [11, 12]. M protein consists of an N-terminal variable domain and a conserved C-terminal domain. Expression of M protein on the bacterial surface is important for GAS virulence, as this mediates the adhesion and internalization of GAS into host cells [13–16]. The N-terminal domain of M protein can bind to serum complement regulator C4b-binding protein (C4BP), which helps GAS avoid phagocytic killing by interfering with host complement activation [17]. The 26-valent recombinant M protein vaccine is designed to induce antibodies that target the N-terminal sequences of the most common *emm* types of GAS in developed countries [7–9]. Furthermore, a synthetic peptide-based vaccine that targets the conserved C-terminal domain of M proteins also achieved protective immunity against different *emm* types of GAS infection [18, 19]. However, the repeat immunization of recombinant M5 protein, which contains the N-terminal and C-terminal domains, induces autoimmune response against cardiac tissues and causes rheumatic heart disease [20].

In addition to M protein, virulence factors, such as fibronectin-binding proteins (FnBP), streptococcal pyrogenic exotoxin B (SPE B), and streptolysin S (SLS), are also involved in the pathogenesis of GAS [21, 22]. The fibronectin-binding proteins mediate extracellular matrix binding and help GAS internalization into cells [14]. One group of the fibronectin binding proteins, including PrtF1 (Sfb1), PrtF2, FbaB (PrtF2-like) and serum opacity factor, contain a series of tandem fibronectin-binding repeats (FnBR) within their sequences [23, 24]. More than 50% of clinical group A streptococcal isolates carry the *sfb1* gene [25, 26]. A previous report indicated that immunization of mice with Sfb1 induces a protective immunity to lethal group A streptococcal infection [27]. Sfb1 and M protein can bind the Fc-motif of immunoglobulin to inhibit antibody function and classical complement activation [28, 29]. The mutation of Sfb1 and M proteins of GAS is able to reduce bacterial binding and internalization into epithelium cells and endothelial cells [30].

In contrast to M protein and fibronectin-binding proteins, which are expressed on the bacterial surface, SPE B is the most highly secreted protein in almost all strains of group A streptococcus [31]. SPE B cleaves and inactivates the cationic antibacterial peptide cathelicidin LL-37 and chemokines [32, 33], and it also inhibits complement-mediated GAS killing by degrading complement C3 and the C5b-C9 complex [34, 35]. Furthermore, SPE B degrades the complement regulators properdin, C1-esterase inhibitor, and IgG to interfere with the host immune defense system [35–38]. In our previous study, we found that the C-terminal motif of SPE B is a major binding site for human IgG and complement C3. Immunization with the C-terminal motif of SPE B effectively protects mice from M49 strain GAS-induced death. However, the protective effect is not significant against invasive M1 strains of GAS infection [38, 39]. In addition, SPE B also degrades M proteins, PrtF1, streptokinase, streptolysin O, and C5a peptidase of GAS, indicating that it may modulate the effects of streptococcal virulence factors during GAS infection [40]. Reports indicate that mutations with CovRS decreases SPE B production and that this is associated with severe invasive GAS infection [41, 42]. Although the repression of SPE B expression is considered to be an advantage for CovRS mutants during invasive infection, the mutation of CovRS not only causes down-regulation of SPE B, but also up-regulates the expression of NAD glycohydrolase, SLS, and *hasABC* [43–45].

Streptolysin S is an important virulence factor of GAS and is associated with GAS-induced tissue injury and mortality [46]. SLS inhibits neutrophil extravasation and induces cell death during the first few hours after GAS inoculation [47, 48]. Our previous report also indicates that SLS synergistically acts with SPE B to enhance tissue damage and mortality in GAS-infected mice [49]. Although the role of SLS is known, it is difficult to naturally induce the production of anti-SLS antibodies in GAS infected individuals because of the low immunogenicity of SLS [50]. In the present study, a gene encoding for a poly-epitopes recombinant FSBM protein (rFSBM) was synthesized, cloned, and expressed, and its protein product was characterized. The recombinant FSBM protein contained four different epitopes selected from four GAS virulent factors, including the FnBR domain of Sfb1 [23, 51], the C-terminal immunogenic segment of SLS [50], the C3-binding motif of SPE B [39], and the C-terminal conserved segment of M protein [18, 19]. Immunization with the rFSBM protein effectively protected mice against lethal infection with different *emm*-types of group A streptococcal bacteria, including invasive *emm1* and *emm12* strains of GAS. *In vitro* experiments indicated that the opsonization of group A streptococcal bacteria was enhanced by anti-rFSBM serum. These findings suggest that the polyvalent recombinant FSBM protein could be applied as a vaccine to protect against group A streptococcal infection.

## Materials and methods

### Bacterial strains

The clinically isolated *emm1*-, *emm4*-, *emm6*-, *emm12*-, *emm22*-, and *emm49*-types of group A streptococcus, the *speB* mutant strain SW507, and the *emm6* deletion mutant GAS were provided by Professor J. J. Wu of the Department of Medical Laboratory Science and Biotechnology of National Cheng Kung University. The bacteria were cultured in THY broth (Todd-Hewitt broth containing 2% yeast extract) overnight at 37°C and then subcultured in fresh THY broth for another 5 hours. The *speB* mutant SW507 was cultured in THY broth containing 50 μg/ml of kanamycin, as previously reported [52]. The concentration of the bacteria was determined with a spectrophotometer (Beckman Instruments, Somerset, NJ) by measuring the OD_600_.

### Reagents and antibodies

Streptolysin S, Freund's adjuvant, and porcine cardiac myosin protein were purchased from Sigma-Aldrich. The synthetic peptides, which contain amino acid residues 373 to 409 of Sfb1, were synthesized by Genemed Biotechnologies, Inc. Rhodamine-conjugated fibronectin was obtained from Cytoskeleton, Inc. The squalene-based oil-in-water adjuvant AddaVax was purchased from InvivoGen. The mouse polyclonal antibodies recognizing the C-terminal epitope of SPE B (anti-SPE B serum) were generated, as previously reported [52]. Horseradish peroxidase (HRP)-conjugated goat anti-mouse IgG and fluorescein isothiocyanate (FITC)-conjugated goat anti-mouse IgG were obtained from Invitrogen.

### Synthesis, cloning and expression of recombinant FSBM

The sequences of Sfb1, SLS, SPE B and M1 protein were retrieved from the GenBank database, and four immunogenic epitopes GMSGQTTPQVETEDTKEPGVLMGGQ, FSIATGSGNSQGGSGSYTPGKC, ALGTGGGAGGFNGYQSAVVGIKP, and QAEDKVKQSREAKKQVEKALKQLEDKVQ, of these streptococcal virulence factors were selected. Based on the DNA sequences of these epitopes, a 357-bp synthetic gene, *fsbm*, was designed and synthesized (Genemed Biotechnologies, Inc.). The synthetic *fsbm* gene was then cloned into a pET-24a expression vector (Novagen), and the sequence was confirmed by DNA sequencing. The pET24a-*fsbm* plasmid was transformed into *E*. *coli* BL21 (DE) pLysS competent cells and selected using 50 μg/ml kanamycin. The recombinant protein, rFSBM, was induced by 0.5 mM IPTG and purified using Ni^2+^-chelating chromatography (GE Healthcare). The purified recombinant protein was further examined by SDS-PAGE and Western Blot analysis using mouse anti-SPE B serum (1: 2,500) to verify the expression of the SPE B epitope within the recombinant FSBM protein.

### Immunizations and infection models

Seven- to 8-week old female BALB/cByJNarl mice were purchased from the National Laboratory Animal Center in Taiwan. They were maintained on standard laboratory chow and water *ad libitum* in the animal center at I-Shou University. Twenty-five micrograms of recombinant FSBM protein were intraperitoneally inoculated into mice either alone or combined with complete Freund’s adjuvant (rFSBM/CFA) or squalene-based adjuvant AddaVax (rFSBM/AddaVax), and then, the mice received 2 boosters of the rFSBM vaccination (alone or in adjuvant combination) at one-week intervals. The formulation of AddaVax is squalene-based oil-in-water nano-emulsion, which is similar to the licensed human flu vaccine adjuvant MF59. For pathologic analysis, rFSBM-immunized mouse kidney and heart tissues were collected and fixed in a 10% neutral-buffered formalin solution after being sacrificed by carbon dioxide (CO_2_) inhalation. Then, the tissues were embedded in paraffin. Five-μm-thick tissue sections were stained with hematoxylin and eosin (H&E) and then evaluated by histological analysis. Mouse serum creatinine (CRTN) and blood urea nitrogen (BUN) were measured using the Beckman Image System (Beckman Coulter, Brea, CA, USA) in the Department of Pathology of the E-Da Hospital. After immunization, groups of 4 to 5 mice were inoculated with 1–2 × 10^8^ colony forming units (CFU) of *emm1*, *emm4*, *emm12*, or *emm22* strains of group A streptococcus through the subcutaneous or intraperitoneal route. The mice survival rates as well as the tissue lesions were monitored twice a day for a total of 14 days, the degrees of skin lesion were photographed and measured using the ImageJ software (National Institutes of Health, Maryland, USA). The air pouch exudate of *emm1* type GAS-infected mouse was collected by injecting 1 ml of sterile PBS into the air pouch and aspirated using a syringe at 48 h after infection. Thereafter, the air pouch exudates were serially diluted and plated onto TSBY agar plates to quantitate the bacterial numbers. The mice that manifested clinical signs, including dyspnea, cyanosis, complete anorexia, and 20% weight loss, were humanely sacrificed using CO_2_ euthanasia to avoid unnecessary suffering. At the end point of the experiment, all the mice were sacrificed by CO_2_ euthanasia and also received cervical dislocation to ensure death. All procedures, care, and handling of the animals were reviewed and approved by the Institutional Animal Care and Use Committee at I-Shou University. (IACUC-ISU-101026).

### ELISA analysis

The levels of rFSBM-specific IgG of immunized sera collected from the rFSBM-, rFSBM/AddaVax-, rFSBM/CFA-immunized mice or adjuvant-only controls were determined using an enzyme-linked immunosorbent assay (ELISA). Microtiter plates were coated with 0.1 μg of rFSBM protein per well and incubated with the serial diluted sera at 37°C for 30 min. After washing with 0.05% Tween-20 phosphate-buffered saline (PBST), 0.2 ml of HRP-conjugated goat anti-mouse IgG antibody (1: 5,000) was added and incubated for 1 h at room temperature. The antibody titers were determined by reading the absorbance at 650 nm with Multiskan EX Microplate Reader (Thermo Fisher) after development with tetramethylbenzidine (Vector Laboratories). For antigenic specificity analysis, microtiter plates were coated with 0.1 μg of purified SPE B, commercial streptolysin S, synthetic peptide of Sfb1, porcine heart myosin protein or the pepsin-cleaving M protein (pepM), and then an ELISA detection procedure was conducted with rFSBM-immunized serum or the CFA adjuvant-only control serum, as described above. SPE B was purified as described in our previous report [52]. The pepM proteins were obtained from *emm1*, *emm4*, *emm6*, *emm12*, *emm22* strains or *emm6*-deficient mutant strains of group A streptococcus by pepsin cleaving, as described in previous reports [53].

### Flow cytometry and western blotting

Different *emm* strains of GAS and the *emm6*-deletion mutant were diluted to 10^5^ CFU with sterile phosphate-buffered saline (PBS) and mixed with an equal volume of anti-rFSBM serum or CFA adjuvant-only control serum dilutions at 37°C for 30 min to determine the levels of the M protein-specific opsonic antibodies. The serum-treated bacteria were washed with PBS and then incubated with 50 μl of FITC-conjugated goat anti-mouse IgG antibody for 30 min at room temperature. After washing with PBS, the serum-treated bacteria were suspended in 0.5 ml of PBS and analyzed using the mean fluorescence intensity (MFI) determined with the FACSCalibur cytometer (Becton Dickinson). In a fibronectin-binding assay, the sera-treated *emm1* type GAS was further incubated with 50 μl of rhodamine-conjugated fibronectin for 1 h at room temperature and then analyzed by flow cytometry. In addition, the cardiac myosin protein, the pepM proteins collected from the *emm6* wild type and *emm6* deletion mutant strains, the overnight culture supernatants of *emm1*-type GAS and its isogenic *speB* mutant were separated using SDS-PAGE, blotted to polyvinylidene difluoride (PVDF) membrane, and detected by anti-rFSBM serum.

### Hemolysis assay

A 50-fold dilution of commercial SLS (100 U/ml) was mixed with serially diluted anti-rFSBM or CFA adjuvant-only control serum and then added to a test tube that contained 250 μg/ml of water-soluble cholesterol (Sigma-Aldrich) and 2 × 10^7^ fresh mouse red blood cells. The water-soluble cholesterol could prevent the hemolytic activity of streptolysin O that may be contaminated in the commercial SLS. After incubation at 37°C for 45 min, the supernatants were collected by centrifugation, and the hemolytic activities were determined by reading the absorbance at 541 nm with an ELISA plate reader. The hemolytic percentages were presented as the absorbance values of the anti-rFSBM serum- or CFA adjuvant-only control serum-treated groups compared with the water-treated red blood cells.

### *In vitro* adhesion and invasion assays

Mouse macrophage RAW 264.7, human lung epithelial A549 cells, and endothelial HMEC-1 cells were provided by Professor Y. S. Lin of the Department of Microbiology and Immunology of National Cheng Kung University. The wild type *emm6* strain JRS4 or the *emm6*-deletion mutant GAS was grown and then opsonized with anti-rFSBM serum, CFA adjuvant-only control serum, or sterile PBS at 37°C for 1 h. For the adhesion assay, RAW 264.7 cells cultured in DMEM containing 5% FCS were seeded in 24-well plates, and then infected with the serum-treated bacteria at a MOI of 25 for 1 h at 37°C. The unbound bacteria were removed by washing with sterile PBS and the total number of bacteria associated with the RAW 264.7 cells were quantified by plating on THY agar plates.

Human epithelial A549 cells and HMEC-1 cells were cultured in DMEM containing 5% FCS and Medium 200 containing low serum growth supplement (Thermo Fisher Scientific), respectively. For the adhesion inhibition assay, A549 cells and HMEC-1 cells were infected with anti-rFSBM serum- or CFA adjuvant-only control serum-treated *emm1* type GAS at a MOI of 20 for 1 h. The unbound bacteria were removed by washing with sterile PBS and the total number of bacteria associated with the cells was quantified by plating on THY agar plates. For the invasion assay, A549 cells and HMEC-1 cells were infected with serum-treated *emm1* type GAS at a MOI of 20 for 1 h. After that, gentamicin (100 μg/ml) was added to kill the extracellular GAS for 1 h, and then the viable intracellular bacteria were quantified by plating on THY agar plates as described previously [54].

### Statistical analysis

Statistical analyses were performed using *t* tests and an ANOVA, and the survival curve comparisons were made using the log-rank test with the Graph Pad Prism software. Differences were considered significant at *P* < 0.05.

## Results

### Expression and characterization of recombinant protein FSBM

**Fig 1:**
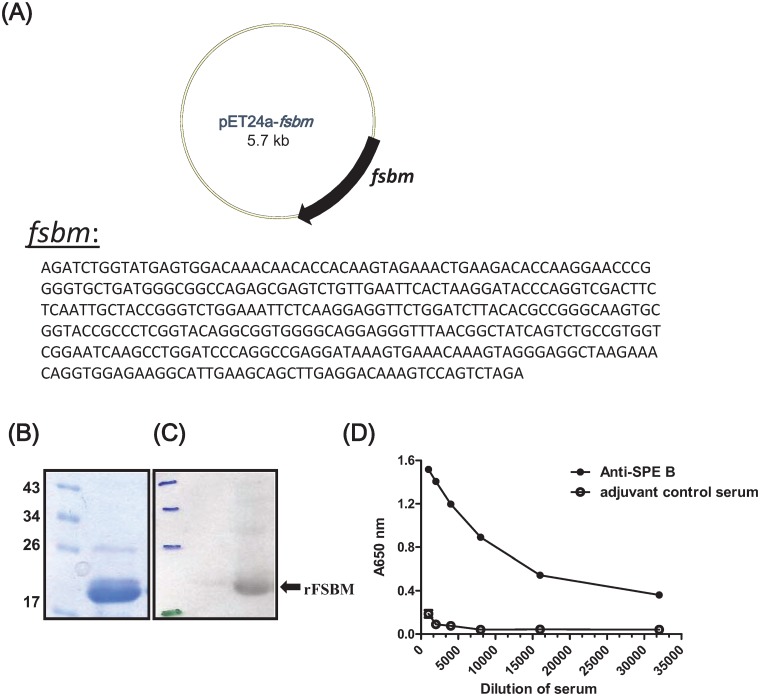
Construction and purification of the recombinant FSBM protein. (A) The map of the pET24a-*fsbm* plasmid and the sequence of the synthetic *fsbm* gene. The plasmid was transformed, and the expression of rFSBM was induced. The recombinant protein was purified as described in Materials and Methods. Purified recombinant rFSBM protein was separated by SDS-PAGE (B) and the antigenic epitope of C-terminal domain of SPE B within rFSBM protein was analyzed by Western blotting (C) and ELISA (D) using the anti-SPE B antibody.

The rFSBM protein, which contains a C-terminal His-tag, was produced by the pET24a-*fsbm* plasmid (Fig 1A)-transformed *E*. *coli*. The rFSBM protein was isolated by affinity chromatography and analyzed by SDS-PAGE. A major band with an apparent molecular mass of 18 kDa was present on the SDS-PAGE gel (Fig 1B). The antigenic epitope of the C-terminal domain of SPE B within rFSBM was further analyzed by Western blotting and an ELISA using the anti-SPE B mouse serum. The Western blotting (Fig 1C) and ELISA (Fig 1D) results both indicated that the recombinant FSBM was recognized by the anti-SPE B mouse serum.

### Measurement of the antibody titers and protective effectiveness after rFSBM vaccination

**Fig 2:**
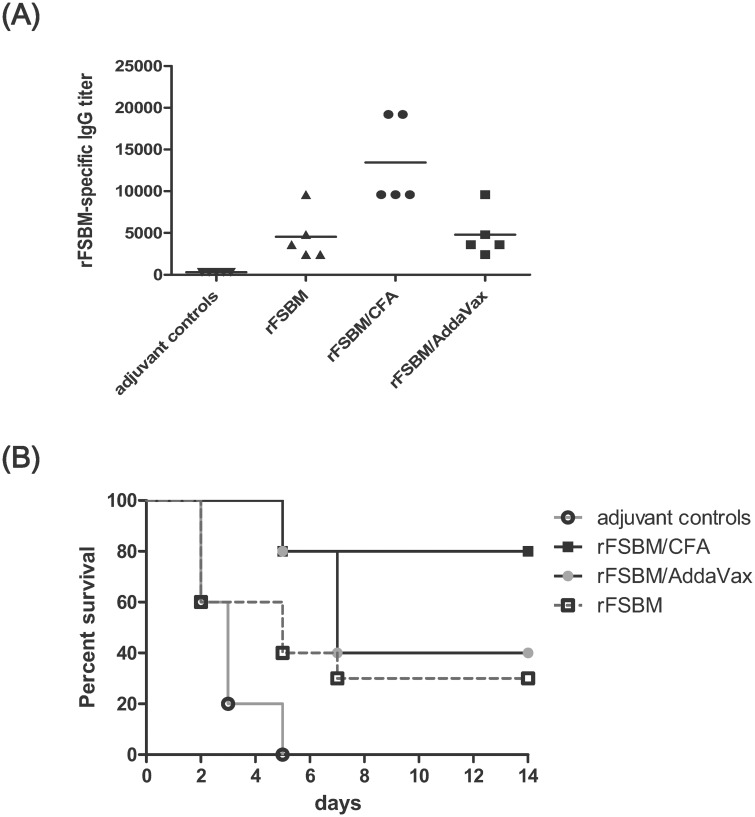
Immunization of recombinant FSBM protein protected mice against invasive *emm1* strain GAS-induced death. (A) The average titers of rFSBM-specific serum IgG collected from BALB/c mice that were immunized with recombinant FSBM proteins alone or rFSBM proteins combined with Freund's Adjuvant or AddaVax. (B) The survival rates of mice immunized with the rFSBM protein combined with different adjuvants were significantly higher than the adjuvant controls, including the mice immunized with CFA adjuvant only or immunized with AddaVax only controls, post subcutaneously challenged with 2 x 10^8^ CFU of *emm1* strain GAS. The survival curves were compared for significance using the log-rank test for the immunized mice compared to the adjuvant-only control groups (*P* < 0.01).

Groups of BALB/c mice were immunized with rFSBM alone, rFSBM/AddaVax, or rFSBM/CFA to determine the effectiveness of rFSBM vaccination against group A streptococcal infection. The sera were collected from the immunized mice 5 days after the secondary booster, and the antibody titers were estimated by an ELISA. The results indicated that the rFSBM/CFA-immunized mice produced higher antibody titers than the other immunized groups. The antibody titers of the rFSBM/AddaVax- and the rFSBM only-immunized mice were similar, which indicated that the immunization of rFSBM without any adjuvant could induce specific anti-rFSBM IgG antibodies (Fig 2A). The H&E staining of heart and kidney sections from rFSBM-immunized mice showed no significant histopathological changes compared with naïve mice (S1 Fig). Furthermore, anti-rFSBM antibody did not cross react with the cardiac myosin heavy chain and myosin light chain in the ELISA and Western blot assays (S2 Fig). In addition, there were no significant differences in the CRTN and BUN levels between the rFSBM-immunized mice (CRTN: 0.44 ± 0.08 mg/dl; BUN: 21.5 ± 2.98 mg/dl) and the naïve control mice (CRTN: 0.42 ± 0.05 mg/dl; BUN: 20.9 ± 2.75 mg/dl). These results demonstrated that immunization of rFSBM did not induce organ specific autoantibodies against the heart and the kidney. The survival rate of the rFSBM/CFA-immunized mice was higher than that of the rFSBM/AddaVax- and the rFSBM-immunized groups after subcutaneous infection with the *emm1* strain GAS, which indicated that an increase in the anti-rFSBM IgG levels was associated with decreased GAS-induced mortality (Fig 2B). These results indicated that the immunization with the rFSBM proteins, alone or combined with adjuvants, was able to induce the production of anti-rFSBM IgG antibodies and effectively protect mice from invasive group A streptococcal infection (Fig 2).

### Characterization of the antigenic specificities of the rFSBM antiserum

**Fig 3:**
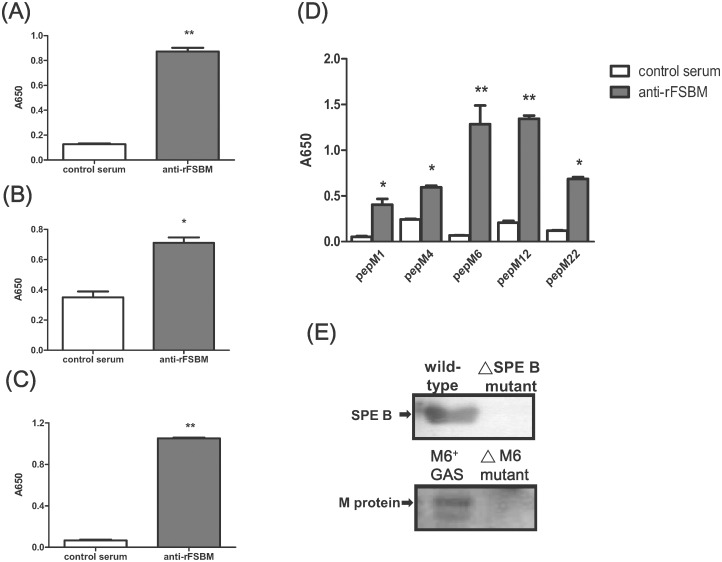
Specific reactivity of the rFSBM–immunized serum with their relative antigens. The rFSBM-immunized serum recognized purified SPE B (A), commercial streptolysin S (B), synthetic peptides of Sfb1 (C), and the pepM proteins collected from different *emm* types of GAS (D). *P < 0.05, **P < 0.01, compared with values determined for the CFA adjuvant only control serum. The SPE B or the pepM proteins purified from the culture medium of wild-type GAS, *speB*-mutant, or the *emm6* deletion mutant were determined by Western blotting (E) as described in Materials and Methods.

**Fig 4:**
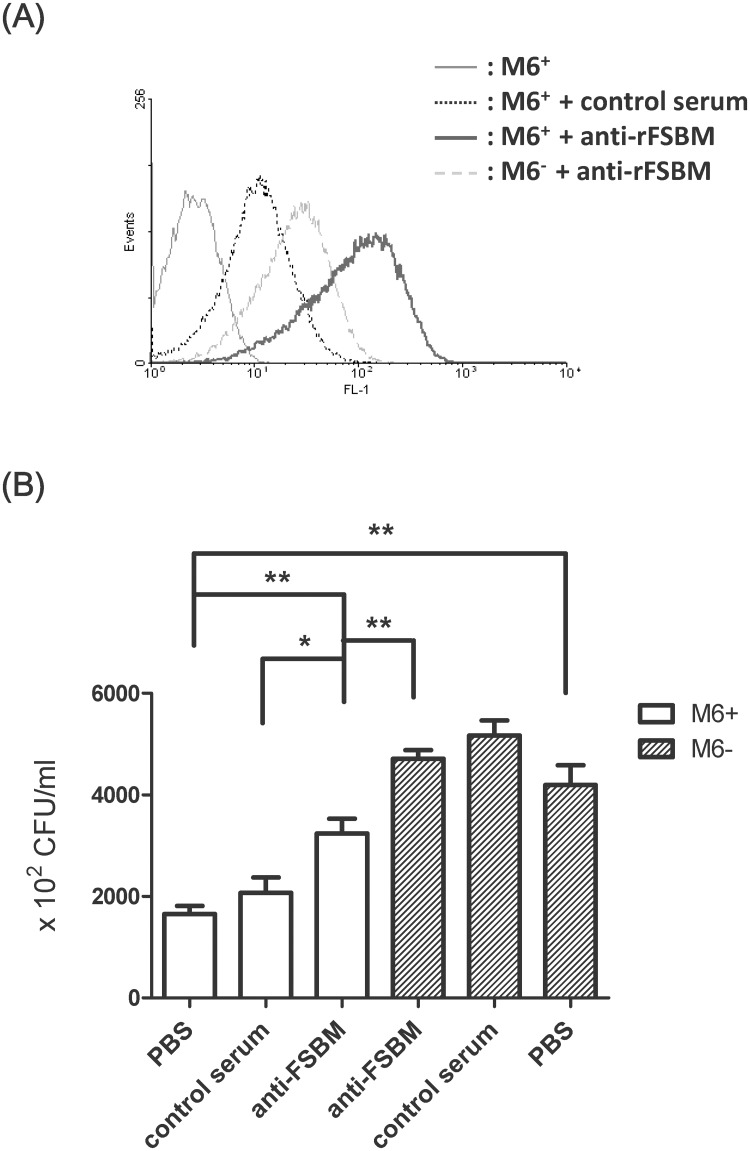
*In vitro* opsonization and macrophage-associated bacteria were enhanced by the rFSBM antiserum. (A) The M protein-specific reactivity of rFSBM-immunized sera was determined by flow cytometry with *emm6* wild type GAS and the *emm6*-deletion mutant, as described in Materials and Methods. (B) Sera from rFSBM-immunized mice or CFA adjuvant-only control mice were incubated with the wild type *emm6*-type GAS or the *emm6*-deletion mutant for 1 h, and then serum-treated GAS was taken to infect RAW 264.7 cells at a MOI of 25 for 1 h. The unbound bacteria were removed by washing with sterile PBS and the total number of bacteria bound with the cells and intracellular bacteria were quantified by plating on THY agar plates as described in Materials and Methods. The macrophage-associated bacterial number of anti-rFSBM serum-treated wild type GAS was increased compared to that of the CFA adjuvant-only control serum-treated GAS. The results of three experiments are shown and expressed as the mean ± SD. *P< 0.05, compared with the values determined for the control serum-treated *emm6* wild type GAS.**P< 0.01, compared with the values determined for the PBS-treated *emm6* wild type GAS or the anti-rFSBM serum-treated *emm6*-deletion mutant.

The antigen specificity of rFSBM-immunized serum was further determined by an ELISA, Western blot, and flow cytometry. Microtiter plates coated with 0.1 μg of SPE B, SLS, pepM proteins, or synthetic peptides of Sfb1 were incubated with rFSBM-immunized serum or CFA adjuvant-only control serum. The results of the ELISA indicated that SPE B, pepM proteins, and synthetic peptides of Sfb1 were effectively recognized by rFSBM-immunized serum at a dilution of 1: 12,000 (Fig 3A, 3C and 3D) while commercial SLS was recognized by rFSBM antiserum diluted to 1: 1,200 (Fig 3B). The Western blotting results also showed that the secreted SPE B and pepM proteins, cleaved from the wild-type GAS, but not from the isogenic mutants, were specifically recognized by the anti-rFSBM serum (Fig 3E). Furthermore, we used a flow cytometric assay to confirm the antigenic specificity of anti-rFSBM serum to GAS. The results showed that the anti-rFSBM serum effectively bound to *emm6* wild type GAS but not its isogenic M protein-deletion mutant strain (Fig 4A). The *emm1*, *emm4*, *emm12*, *emm22*, and *emm49* types of GAS strains were also selected to estimate the M protein-specific reactivity of the rFSBM-immunized serum by means of flow cytometry. The results indicated that the anti-rFSMB antiserum also could bind different types of M proteins of the GAS strains (S3 Fig). These results indicated that the antibodies within the anti-rFSBM serum could specifically bind to the surfaces of *emm1*, *emm4*, *emm6*, *emm12*, *emm22*, and *emm49* types of GAS and may therefore enhance antibody opsonization.

### Analysis of the opsonization activity of anti-rFSBM serum

M protein is known to contribute to the phagocytosis resistance to GAS [17]. To examine the opsonization activity of rFSBM antiserum, we detected macrophage-associated bacterial numbers at 1 h post-GAS infection. As shown in Fig 4B, the association of PBS-treated *emm6*-deletion mutant to RAW264.7 cells (4.19 ± 0.36 × 10^5^ CFU/ml) was higher than those of PBS-treated *emm6* wild type GAS (1.65 ± 0.16× 10^5^ CFU/ml). Moreover, the association of *emm6* wild type GAS to the RAW264.7 cells was significantly enhanced by anti-rFSBM serum treatment (3.24 ± 0.29 × 10^5^ CFU/ml) compared to the adjuvant-only control serum-treated (2.07 ± 0.30× 10^5^ CFU/ml) or the PBS-treated group. These results indicate that the M protein-mediated anti-macrophage association was inhibited by rFSBM antiserum in *emm6* wild type GAS.

### Analysis of the neutralization activities of anti-rFSBM serum to GAS

**Fig 5:**
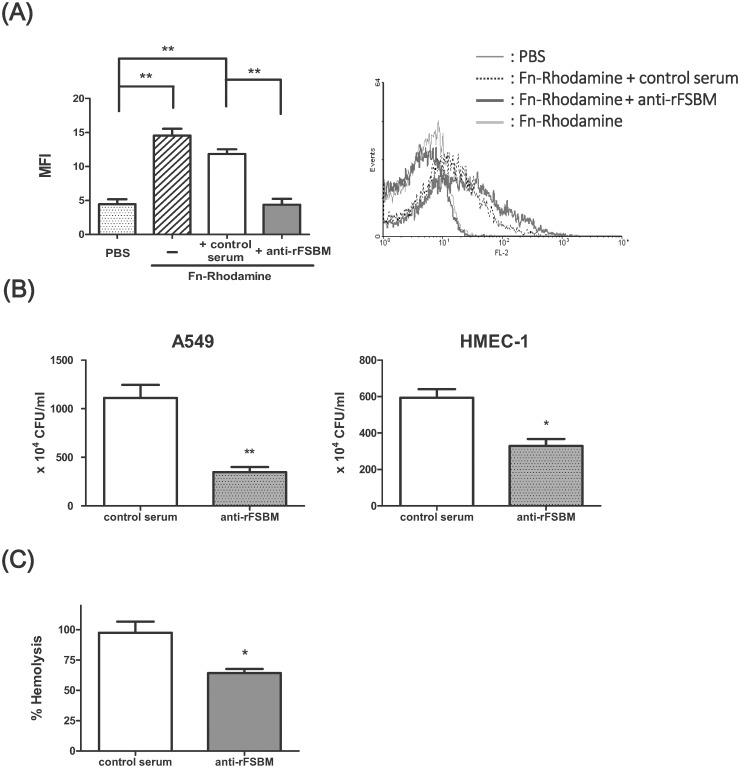
Neutralization activities of anti-rFSBM serum. (A) Competitive inhibition of fibronectin binding by anti-rFSBM serum. Sera from rFSBM-immunized mice or CFA adjuvant-only control mice were incubated with *emm1*-type GAS for 1 h and then added to rhodamine-conjugated fibronectin for another 1 h. The MFI of the GAS was measured using a flow cytometer. (B) The rFSBM-immunized serum or the CFA adjuvant-only control serum was incubated with *emm1*-type GAS and then used to infect A549 or HMEC-1 cells. The unbound bacteria were removed by washing with sterile PBS and the total number of bacteria bound with the cells was quantified by plating on THY agar plates. *P< 0.05, **P< 0.01, compared with the values determined for the CFA adjuvant-only control serum. (C) The anti-rFSBM serum or CFA adjuvant-only control serum was incubated with streptolysin S and mouse RBC for 1 h, and the percentage of hemolysis was measured using an ELISA reader, as described in the Materials and Methods. *P< 0.05, compared with the values determined for the control serum.

M proteins and fibronectin-binding proteins both play an important role in cell adhesion and invasion during group A streptococcal infection. Our ELISA results indicated the anti-rFSBM serum could recognize the pepM proteins of several *emm* types of GAS and the fibronectin-binding motif of Sfb1 (Fig 3). A competitive inhibition assay confirmed that the rFSBM antiserum, but not the CFA adjuvant-only control serum, effectively inhibited the binding of rhodamine-conjugated fibronectin to *emm1*-type GAS (Fig 5A). In the antibody neutralization assays, A549 cells or HMEC-1 cells were infected with anti-rFSBM serum or CFA adjuvant-only control serum-treated *emm1*-type GAS for 1 h, and the adhesion of GAS to the both cells was determined by counting the number of bacteria bound with cells. As shown in Fig 5B, the adhesion of GAS to the A549 cells and HMEC-1 cells was significantly inhibited by anti-rFSBM serum. Furthermore, the intracellular bacterial numbers of GAS-infected A549 cells or GAS-infected HMEC-1 cells were reduced in the anti-rFSBM serum group compared to the CFA adjuvant-only control serum group, which indicated that the invasion of GAS was also blocked by antibodies within the anti-rFSBM serum (S4 Fig). The serum from rFSBM-immunized mice also reduced the SLS-induced hemolysis of mouse red blood cells, while the serum from the control mice did not prevent SLS-mediated hemolysis (Fig 5C). These results indicated that the anti-rFSBM serum not only neutralized the adhesion activity of GAS molecules to their ligands, but also decreased the hemolytic activity of streptolysin S.

### Immunization of recombinant FSBM proteins protected the mice against different *emm* type GAS-induced tissue damage and mortality

**Fig 6:**
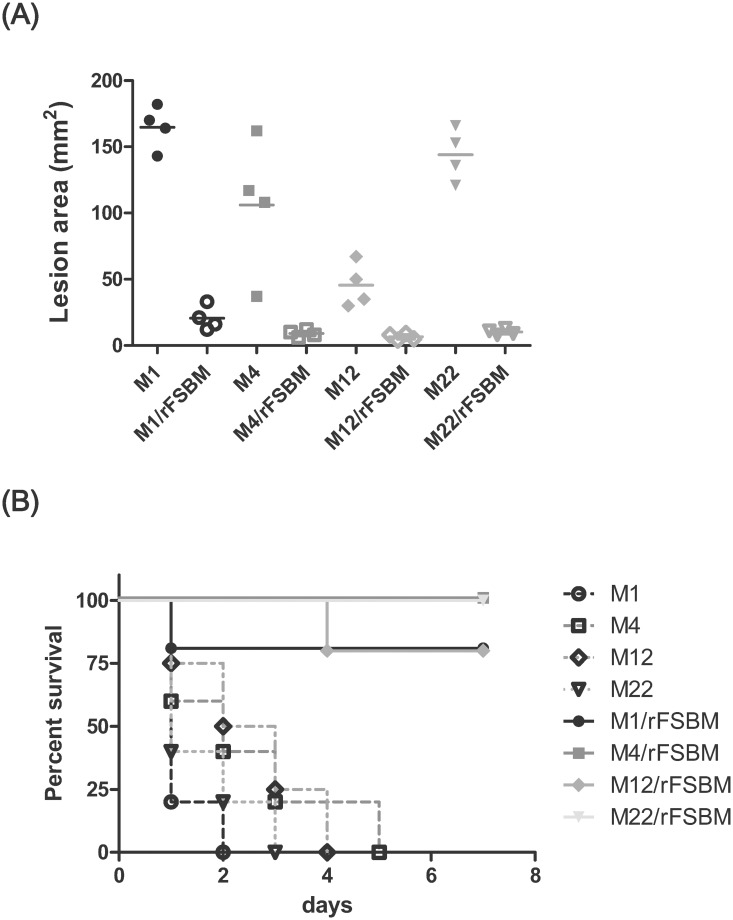
Immunization with recombinant FSBM protein protected mice against different *emm* type GAS-induced tissue damage and mortality. The severity of the skin lesions (A) and the mortality (B) in the rFSBM-immunized mice were significantly decreased compared to the control mice after air pouch inoculation with 2 x 10^8^ CFU of different *emm* types of GAS (A) or intraperitoneal infection with 1 x 10^8^ CFU of different *emm* types of GAS (B). The survival curves were compared for significance using the log-rank test for the immunized mice versus the control group (*P*< 0.01).

Our *in vitro* results indicated that the anti-rFSBM serum recognized four important virulence factors of GAS, including M proteins, Sfb1, SPE B, and SLS. Moreover, the anti-rFSBM serum also mediated the neutralization of hemolytic activity of SLS and affected GAS adhesion to cells. A mouse air-pouch infection model was used to evaluate the protective effects of rFSBM vaccination against different *emm* strains of GAS infection. The air-pouch inoculation of 2 × 10^8^ CFU of *emm1* and *emm12* caused 80% and 60% mortality of mice, respectively (data not shown). The survival rates of *emm1*- and *emm12*-type GAS infected mice were significantly improved after rFSBM vaccination. The immunization of rFSBM increased survival rates of *emm1*- and *emm12*-type GAS infected mice from 20% and 40%, respectively, up to 100%. The skin lesions of the rFSBM-immunized mice (21 ± 8 mm^2^) were smaller compared to the lesions of the control mice (165 ± 14 mm^2^) at 48 h post *emm1*-type GAS infection. At the same time, the number of bacteria in the air pouch exudates of the rFSBM-immunized mice (1.4 ± 0.7 × 10^6^ CFU/ml) was significantly lower than the bacterial number (2.7 ± 1.4 × 10^7^ CFU/ml) of the CFA adjuvant-only control mice. As for the *emm4* or *emm22* strains, the inoculation of 2 × 10^8^ CFU of GAS into the air pouch did not cause mouse death, but it did cause skin lesions around the air pouch. The immunization of rFSBM proteins significantly reduced the area of the skin lesions after the air pouch inoculation with 2 × 10^8^ CFU of *emm1*, *emm4*, *emm12*, or *emm22* strains (Fig 6A). Furthermore, an intraperitoneal infection model was applied to estimate the improvement of the survival effect of rFSBM vaccination. The rFSBM-immunized mice had a significantly increased survival rate (80–100%) compared to the control mice (0%) after intraperitoneal infection with these four *emm* strains (Fig 6B). These findings suggest that the immunization with rFSBM effectively protected the mice against lethal infection with different *emm* types of GAS, even the invasive *emm1* strain of GAS.

## Discussion

Our previous report found that a combinative vaccination of synthetic peptides derived from the C-terminal epitope of streptolysin S and the C3-binding motif of SPE B can elicit a specific immune response against GAS infection. Although a recombinant M protein vaccine against the N-terminal variable residues of 26 different serotypes of GAS has entered clinical trials, it did not target other virulence factors of GAS. In this study, we synthesized, expressed, and purified a recombinant FSBM protein that contained a C-terminal conserved segment of M protein, the FnBR domain of Sfb1, the C3-binding motif of SPE B, and an immunogenic domain of SLS. The immunization of the recombinant FSBM protein successfully induced antibodies against SPE B and SLS (Fig 3A and 3B).

During the early period of GAS infection, host and bacterial proteins are cleaved by SPE B, which mediates host tissue damages and bacterial dissemination [55]. However, SPE B not only degrades host proteins but also cleaves streptococcal surface proteins, which indicates that it may modulate the effects of streptococcal virulence factors such as M protein and PrtF1 during GAS infection [40, 43–45]. Reports also indicate that CovRS mutants isolated from severe invasive *emm1*-type GAS infection are associated with the down-regulation of SPE B expression of GAS [56–58]. All the GAS strains used in our study express SPE B *in vitro*, and the *emm1*-type GAS strain carries wild-type *covR*, *covS* as well as *rgg* genes [59, 60]. A recent study indicated that no SPE B-negative variants were detected from mice after cutaneous infection with the *emm3* and *emm28* GAS, and that about 10% of GAS isolates that recovered from mice after cutaneous infection with the *emm12* GAS were SPE B-negative with CovS mutation compared with the parent strain [61]. Although not all GAS strains express SPE B, SPE B plays an important role in GAS-induced local tissue damage [49, 55]. Our air pouch infection model showed that the skin lesions were significantly reduced in the rFSBM-immunized mice (Fig 6A), indicating that the SPE B activity was inhibited by anti-rFSBM antibodies *in vivo*.

The examination of the effectiveness of the anti-rFSBM serum recognized these four virulent factors and found that the anti-rFSBM serum recognized the SPE B, Sfb1, and pepM proteins at a dilution of 1:12,000, which was 10-fold higher than that required to recognize SLS. In the GAS-infected individual, anti-SLS antibodies were not easily induced because of the weak immunogenicity of the SLS epitope [50]. In our previous report, we indicated that the conjugation of the SLS epitope with keyhole limpet hemocyanin (KLH) is required to induce enough anti-SLS antibodies and protective immunity to prevent tissue damage and mortality in GAS-infected mice [39]. In this study, we found that even without conjugation of the SLS epitope with KLH, anti-rFSBM serum still decreased SLS-mediated hemolysis (Fig 5C). These results indicated that the conjugation of the SLS epitope with epitopes of other virulence factors within rFSBM could enhance its immunogenicity and successfully induce neutralizing antibodies against SLS after rFSBM vaccination.

Clinically-isolated GAS strains are differentiated into class I and class II by the M protein C-repeat region-specific antibodies. The class I strains of GAS are further classified as *emm* patterns A-D based on the 5’ sequences of the *emm* genes. The strains with *emm* patterns A-C are associated with pharyngeal GAS infections, and the *emm* pattern D strains are associated with cutaneous infection. The *emm* pattern E strains that belong to class II GAS strains are isolated from pharyngeal and cutaneous sites [62, 63]. A poly-valance M protein vaccine successfully induces protective immunity against 26 different *emm* types of GAS infection [7, 8]. In this study, immunization with the recombinant FSBM protein, which contains an epitope from the conserved segment of the M proteins, successfully induced antibodies to recognize different *emm* types of GAS. The results of the ELISA and flow cytometry analyses showed that the *emm1*, *emm4*, *emm6*, *emm12*, *emm22*, and *emm49* types of GAS were recognized by FSBM antiserum (Fig 3D and S3 Fig). The anti-rFSBM serum recognized both the strains with *emm* patterns A-C (*emm1*, *emm6*, and *emm12*) and the *emm* pattern E strains (*emm4*, *emm22*, and *emm49*), which means the immunization with the FSBM protein could provide protection against both class I and class II GAS strains.

The *emm1*, *emm4*, *emm12*, *emm28*, and *emm89* types of GAS are the most common invasive strains isolated from patients in Europe and the Pacific region [1, 3, 5]. However, the prevalent *emm* types of GAS isolated from patients with invasive soft tissue infection or streptococcal toxic shock syndrome in Taiwan are *emm*1, *emm4*, *emm11*, *emm12*, and *emm102* types [11, 12]. *In vitro*, the M-protein specific opsonization of GAS was enhanced by anti-rFSBM serum (Fig 4B), and *in vivo* protection against intraperitoneal *emm1*, *emm4*, *emm12*, and *emm22* types of GAS infection was achieved after rFSBM vaccination (Fig 6B). Although the mortality and severity of the skin lesions of *emm1* GAS-infected mice were higher than other *emm* types GAS-infected groups after air pouch inoculation, the immunization of rFSBM proteins significantly decreased the mortality and skin lesions in *emm1* GAS-infected mice (Figs 2B and 6A). These results indicated the immunization of the recombinant FSBM protein could elicit a cross-*emm* type protective response against infection of clinical invasive strains of GAS.

In addition to the conserved motif of M proteins, the recombinant FSBM protein contained a FnBR domain of Sfb1. The streptococcal PrtF2, FbaB, and serum opacity factor all contain FnBR domains, which indicates that the anti-rFSBM serum may recognize other FnBR-containing proteins expressed on the surface of GAS. The *emm4*, *emm6*, and *emm12* types of GAS used in this study carried the *sfb1* gene (data not shown). Although the *sfb1* gene is not found in the genome of the *emm1* type GAS strain A20 used in this study [59], the M1 and M6 protein also mediate the fibronectin binding and help the entry of GAS into epithelial cells [14]. The results of flow cytometry analysis indicated that the anti-rFSBM serum could interfere with the binding of rhodamine-conjugated fibronectin to *emm1* type GAS (Fig 5A). The MFI of the anti-rFSMB serum treated *emm6*-deficient mutant GAS was significantly higher than the MFI of the CFA adjuvant-only control serum, which may be due to the antibodies recognized by the FnBR domains on the bacterial surface (Fig 4A). Several reports have indicated that the fibronectin-binding proteins and M proteins mediate GAS invasion into epithelial cells and endothelial cells [13, 14, 16, 64, 65]. Our *in vitro* studies showed that the GAS adhesion and the intracellular GAS levels within A549 cells and HMEC-1 cells were significantly reduced after anti-rFSBM serum treatment (Fig 5B and S4 Fig), which indicated the anti-FnBR and anti-M protein antibodies within the anti-rFSBM serum could inhibit the adhesion and invasion of GAS into epithelial cells and endothelial cells.

Our present study indicated that the polyvalent recombinant FSBM protein could be applied as a vaccine to protect against group A streptococcal infection. The immunization of rFSBM proteins induced antibodies to recognize streptococcal surface M proteins, Sfb1, and the neutralizing antibodies against streptolysin S and SPE B simultaneously. The presence of the similar streptolysin S gene, Sfb1 gene, M protein gene, and M-like protein gene has been detected in *Streptococcus dysgalactiae* subsp. *dysgalactiae* and *Streptococcus dysgalactiae* subsp. *equisimilis* [66]. Our preliminary flow cytometry analysis indicated that the anti-rFSBM serum could bind to an ATCC strain of *Streptococcus dysgalactiae* subsp. *dysgalactiae*, which is associated with bovine mastitis. *Streptococcus dysgalactiae* subsp. *equisimilis* infects humans to cause clinical symptoms resembling pharyngitis and invasive diseases [67, 68]. Our findings suggested that the vaccination of rFSBM not only protected against group A streptococcal infection but also may be applied to prevent diseases caused by *Streptococcus dysgalactiae* subsp. *dysgalactiae* and *Streptococcus dysgalactiae* subsp. *equisimilis*.

## Supporting information

S1 Fig Histologic analysis of tissues from rFSBM-immunized mice and naïve mice. Hematoxylin and Eosin-stained sections of the heart **(A, C)** and kidney **(B, D)** from rFSBM-immunized mice **(A, B)** showed no significant pathological changes compared to H&E-stained sections of control mice **(C, D)**.
(PDF)

S2 Fig ELISA and immunoblot assays of cardiac myosin proteins with the rFSBM-immunized serum. (A) A group of mice were infected with a sublethal dose of *emm6*-type GAS at day 0 and day 10. On day 21 post-infection, the anti-M6 serum was collected and used as positive control. To determine the cross-reactivity between heart myosin and the rFSBM-antiserum, the microtiter plates were coated with porcine heart myosin protein and then incubated with 1: 100, 1: 500, 1: 1000, 1: 2500, and 1: 5000 dilutions of the rFSBM-immunized serum, the CFA adjuvant-only control serum, or serum collected from the *emm6*-type GAS-infected mouse at 37°C for 1 h. After washing with 0.05% PBST, the HRP-conjugated goat anti-mouse IgG antibody (1: 5000) was added and incubated for 1 h. The cross-reactivity was determined by reading the absorbance at 650 nm after development with tetramethylbenzidine as described in Materials and Methods. The reactivity of anti-serum with porcine myosin was examined by one-way ANOVA. There were significant differences in the anti-M6 serum group versus the anti-rFSBM serum group (*P* < 0.01), the anti-M6 serum group versus the adjuvant-only control serum group (*P* < 0.01), and there was no significant difference between treatments of the anti-rFSBM serum versus the control serum. (B) The purified myosin heavy chain (MyHC) and myosin light chain (MyLC) from porcine heart were separated by SDS-PAGE. (C) The cardiac MyHC and cardiac MyLC did not react with the rFSBM-antiserum (1: 100) according to Western blot analysis. A weak high molecular weight band was observed by Western blotting that may be due to the presence of trace amounts of aggregation of recombinant FSBM protein.
(PDF)

S3 Fig *In vitro* opsonization of different *emm* types of GAS was enhanced by the rFSBM antiserum. The opsonization of *emm1*, *emm4*, *emm12*, *emm22*, and *emm49* types of GAS was significantly enhanced by the rFSBM antiserum treatment compared to the CFA adjuvant-only control serum treatment.
(PDF)

S4 Fig Invasion of *emm1*-type GAS to A549 and HMEC-1 cells was reduced after treatment with the rFSBM antiserum. The rFSBM-immunized serum or the CFA adjuvant-only control serum was incubated with *emm1*-type GAS and then used to infect A549 or HMEC-1 cells. The level of the intracellular bacteria was determined at 2 hours post-GAS infection, as described in Materials and Methods. *P< 0.05, compared with the values determined for the CFA adjuvant-only control serum.
(PDF)
