# Supplementary figures and images for: Immunization with a streptococcal multiple-epitope recombinant protein protects mice against invasive group A streptococcal infection
Source: PLoS One. 2017 Mar 29;12(3):e0174464. doi: 10.1371/journal.pone.0174464 (PMC5371370; doi:10.1371/journal.pone.0174464)

(A)

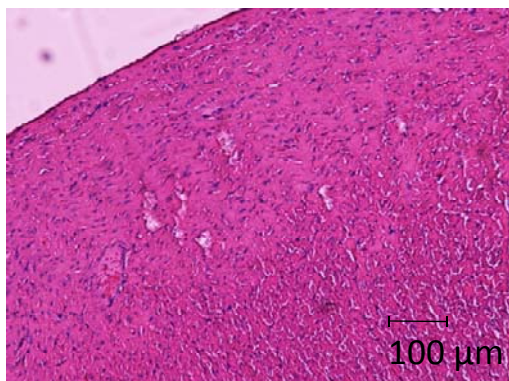

(B)

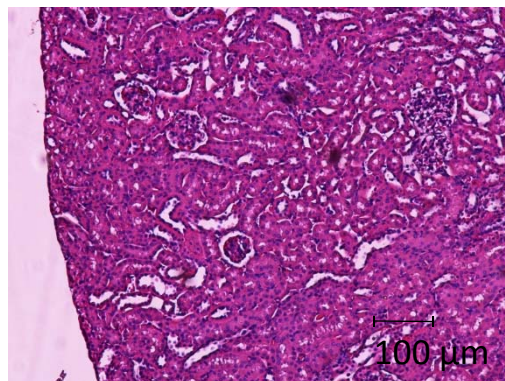

(C)

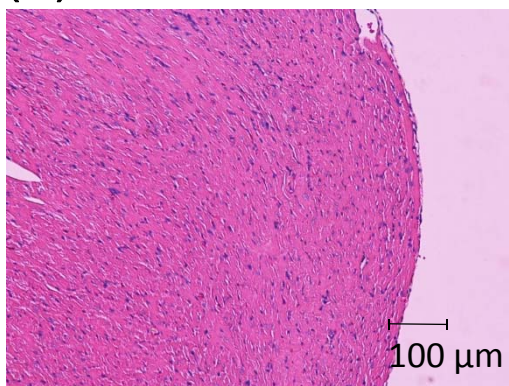

(D)

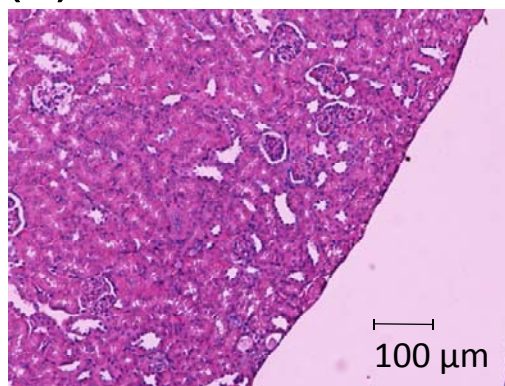

**S1 Fig.**

Supplement: S1 Fig — Hematoxylin and Eosin-stained sections of the heart (A, C) and kidney (B, D) from rFSBM-immunized mice (A, B) showed no significant pathological changes compared to H&E-stained sections of control mice (C, D). (PDF) [file pone.0174464.s001.pdf]

(A)

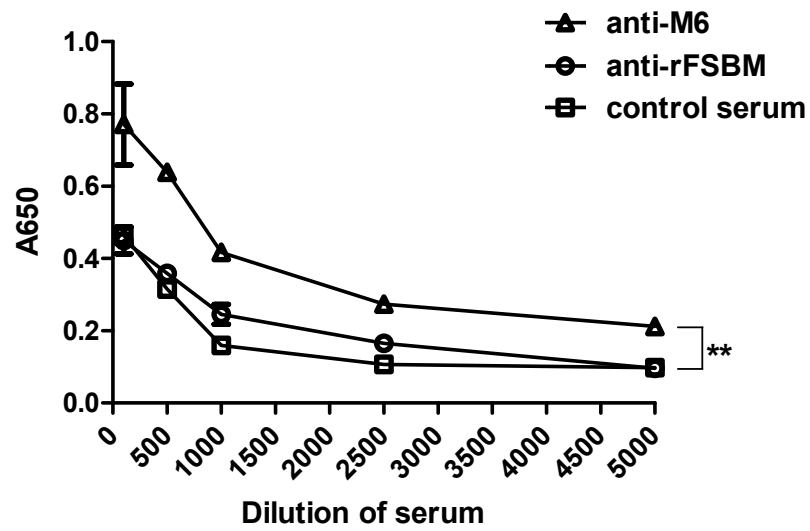

(B)

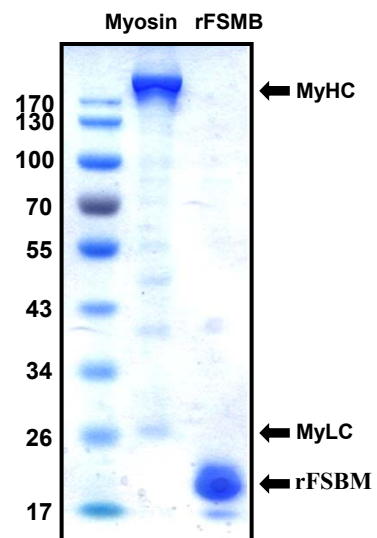

(C)

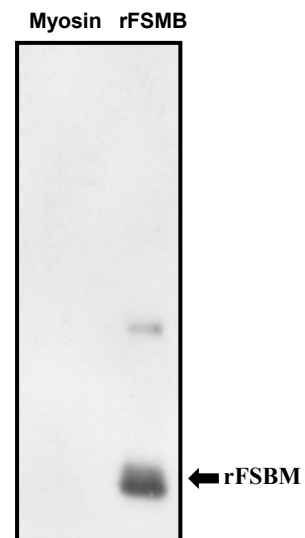

S2 Fig.

Supplement: S2 Fig — (A) A group of mice were infected with a sublethal dose of emm6-type GAS at day 0 and day 10. On day 21 post-infection, the anti-M6 serum was collected and used as positive control. To determine the cross-reactivity between heart myosin and the rFSBM-antiserum, the microtiter plates were coated with porcine heart myosin protein and then incubated with 1: 100, 1: 500, 1: 1000, 1: 2500, and 1: 5000 dilutions of the rFSBM-immunized serum, the CFA adjuvant-only control serum, or serum collected from the emm6-type GAS-infected mouse at 37°C for 1 h. After washing with 0.05% PBST, the HRP-conjugated goat anti-mouse IgG antibody (1: 5000) was added and incubated for 1 h. The cross-reactivity was determined by reading the absorbance at 650 nm after development with tetramethylbenzidine as described in Materials and Methods. The reactivity of anti-serum with porcine myosin was examined by one-way ANOVA. There were significant differences in the anti-M6 serum group versus the anti-rFSBM serum group (P < 0.01), the anti-M6 serum group versus the adjuvant-only control serum group (P < 0.01), and there was no significant difference between treatments of the anti-rFSBM serum versus the control serum. (B) The purified myosin heavy chain (MyHC) and myosin light chain (MyLC) from porcine heart were separated by SDS-PAGE. (C) The cardiac MyHC and cardiac MyLC did not react with the rFSBM-antiserum (1: 100) according to Western blot analysis. A weak high molecular weight band was observed by Western blotting that may be due to the presence of trace amounts of aggregation of recombinant FSBM protein. (PDF) [file pone.0174464.s002.pdf]

***emm1* GAS**

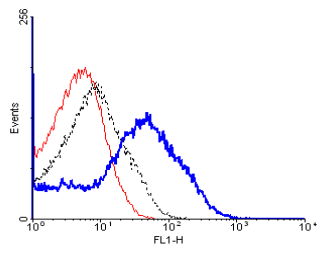

***emm4* GAS**

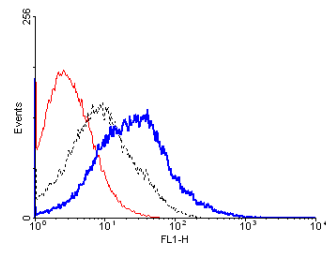

***emm12* GAS**

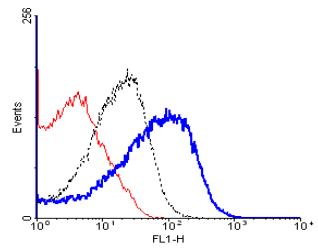

***emm22* GAS**

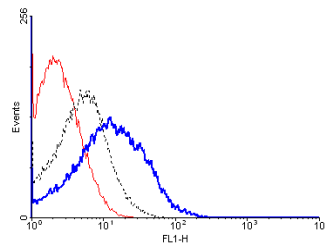

***emm49* GAS**

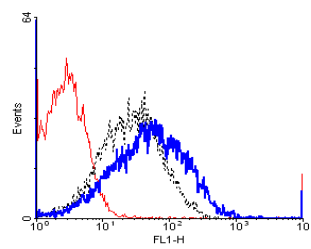

----- : GAS + control serum  
— : GAS + anti-FSBM  
— : GAS

**S3 Fig.**

Supplement: S3 Fig — The opsonization of emm1, emm4, emm12, emm22, and emm49 types of GAS was significantly enhanced by the rFSBM antiserum treatment compared to the CFA adjuvant-only control serum treatment. (PDF) [file pone.0174464.s003.pdf]

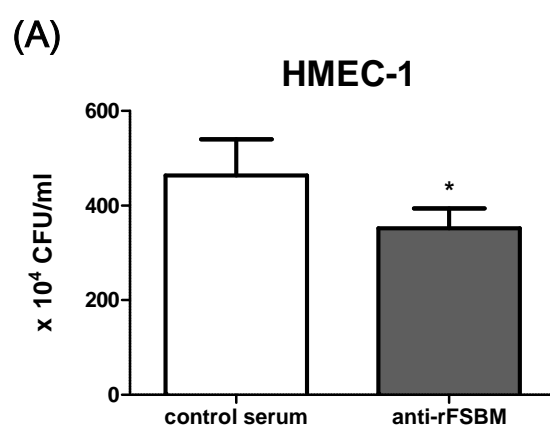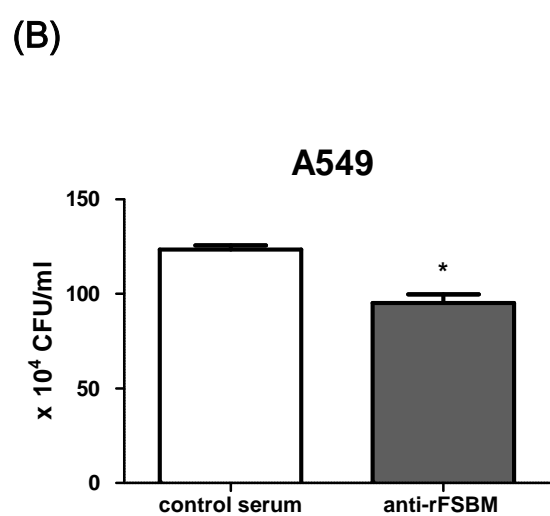

**S4 Fig.**

Supplement: S4 Fig — The rFSBM-immunized serum or the CFA adjuvant-only control serum was incubated with emm1-type GAS and then used to infect A549 or HMEC-1 cells. The level of the intracellular bacteria was determined at 2 hours post-GAS infection, as described in Materials and Methods. *P< 0.05, compared with the values determined for the CFA adjuvant-only control serum. (PDF) [file pone.0174464.s004.pdf]
